# Delta neutrophil index as a prognostic marker in emergent abdominal surgery

**DOI:** 10.1002/jcla.22895

**Published:** 2019-04-15

**Authors:** Jae Seung Soh, Sang‐Woo Lim

**Affiliations:** ^1^ Department of Internal Medicine University of Hallym College of Medicine Hallym University Sacred Heart Hospital Anyang Republic of Korea; ^2^ Department of Colorectal Surgery University of Hallym College of Medicine Hallym University Sacred Heart Hospital Anyang Republic of Korea

**Keywords:** acute abdomen, acute peritonitis, delta neutrophil index, mortality, predictive value

## Abstract

**Background:**

Delta neutrophil index (DNI) is the fraction of circulating immature granulocytes provided by a routine, complete blood cell analyzer. It is known to be a useful prognostic marker of sepsis. The aim of this study was to evaluate the role of DNI in the diagnosis and prognosis of patients who had undergone emergent surgery for an acute abdomen.

**Methods:**

A total of 694 patients who had visited the emergency room for acute abdominal pain and undergone emergent abdominal surgery from May 2015 to September 2016 were retrospectively reviewed. Clinical characteristics, laboratory findings on the day of hospital visit, hospital stay, postoperative complications, and 30‐day mortality were investigated.

**Results:**

In the analysis of patients who had undergone an operation for acute peritonitis, the DNI was a good predictor for predicting 30‐day mortality rate (area under the curve [AUC]: 0.826). It was not inferior to other laboratory values, including activated partial thromboplastin time (AUC: 0.729), C‐reactive protein (AUC: 0.727), albumin (AUC: 0.834), prothrombin time (AUC: 0.816), and creatinine (AUC: 0.837) known to be associated with sepsis. Patients with high DNI displayed higher incidence of bacteremia and sepsis, longer hospital stay, higher postoperative complication rate, and higher 30‐day mortality rate than patients with low DNI. Among patients diagnosed with acute appendicitis, the DNI was a useful marker for differentiating appendiceal perforation.

**Conclusion:**

The DNI was a practical and useful marker for predicting the prognosis of patients who needed emergent abdominal surgery.

## 1 INTRODUCTION

Acute abdomen accounts for 5%‐10% of visits to the emergency department and commonly requires emergent gastrointestinal surgery.^1^ Diagnostic causes of an acute abdomen vary from a relatively mild disease to life‐threatening serious illness. The most common causes of surgical acute abdomen in the emergency department are acute appendicitis with or without perforation, intestinal obstruction, bowel perforation, bowel ischemia, diverticulitis, and hepatobiliary diseases, including acute cholecystitis and cholangitis.^2^ Patients who present with acute abdominal pain require a prompt decision regarding the need for surgical intervention to prevent progression into poor outcome, although some cases require a few hours or days after admission to decide surgical management.

Delta neutrophil index (DNI) is the fraction of circulating immature granulocytes. It has been reported to be a useful prognostic marker of infection or inflammation.^3^, ^4^, ^5^ DNI can be assessed as the difference between leukocyte subfraction determined by cytochemical myeloperoxidase reaction and leukocyte subfraction determined with nuclear lobularity assay by a reflected light beam using an automated blood cell analyzer. It is included in routine, complete blood count (CBC) tests. Along with inflammatory serologic markers, including white blood cell (WBC) counts, C‐reactive protein (CRP), and procalcitonin, DNI serves as a diagnostic tool that can predict mortality in patients with sepsis, disseminated intravascular coagulation, and bacteremia.^6^, ^7^, ^8^ In gastrointestinal diseases, increased DNI values are independently associated with mortality in patients with acute upper gastrointestinal bleeding.^9^ DNI could differentiate perforated appendicitis from non‐perforated appendicitis.^10^ However, the clinical utility of DNI in patients undergoing emergent abdominal surgical procedures for acute abdomen has not been reported yet.

Thus, the objective of this study was to evaluate the role of DNI in diagnosing and predicting the prognosis of patients who underwent emergent surgery.

## 2 MATERIALS AND METHODS

### 2.1 Patients

**Figure 1:**
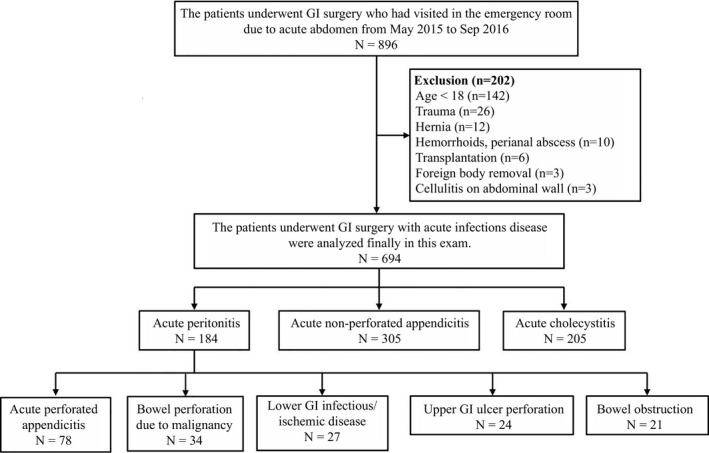
The order of investigations

Medical records of 896 patients who visited the emergency department for acute abdominal pain and underwent emergent abdominal surgery at the Department of Surgery, Hallym University Sacred Heart Hospital, Anyang, Korea, between May 2015 and September 2016 were retrospectively reviewed. Among these patients, 142 patients who were younger than 18 years old, 26 patients who had undergone surgery for trauma, 12 patients who had hernia surgeries, 10 patients who had perianal surgeries, six patients who had liver or kidney transplantation, three patients who had removed foreign bodies on the abdomen, and three patients who had been treated with cellulitis of the abdominal wall were excluded (Figure 1). The remaining 694 patients who had an emergent operation were classified into three groups according to their diseases. Group I included 184 patients who were diagnosed with acute peritonitis. Among them, 78 patients underwent surgical treatments for acute perforated appendicitis. Thirty‐four patients had an operation for cancer perforation (15 with rectosigmoid colon cancer, eight with ascending and transverse colon cancer, four with periampullary cancer, three with stomach cancer, three with hepatocellular carcinoma, and one with small bowel cancer). Fifty‐one patients underwent bowel surgeries because of an infectious or ischemic bowel disease of the lower gastrointestinal tract (n = 27) or an ulcer perforation of the stomach or duodenum (n = 24). The remaining 21 patients underwent operation for bowel obstructive diseases including strangulation, volvulus, intussusception, toxic megacolon, and gallstone ileus. Group II included 305 patients with acute non‐perforated appendicitis, and Group III had 205 patients with acute cholecystitis. The study protocol was approved by the Institutional Review Board (IRB) of Hallym University Sacred Heart Medical Center (IRB 2017‐I055).

### 2.2 Data collection and DNI measurement

Blood tests of all patients were evaluated in the first blood samples collected at the emergency department. CBC counts, chemistry, prothrombin time, activated partial thromboplastin time (aPTT), and CRP were calculated. DNI was examined with a blood cell analyzer (ADIVA 2120i, Siemens Inc, Forchheim, Germany). Clinical parameters such as age, gender, and comorbidity, including hypertension, diabetes, cardiac, renal, and malignant disease, postoperative hospital stay, intensive care unit (ICU) stay, bacteremia, sepsis, postoperative complications, and 30‐day mortality, were collected. For comorbidity, cardiac disease included ischemic heart disease and heart failure and renal disease included chronic kidney disease and glomerulonephritis. Bacteremia was defined as follows: (a) a recognized pathogen (not including organisms known to be common skin contaminants) cultured from one or more blood cultures, and (b) at least one of the following signs or symptoms: fever (>38^°^), chills, and hypotension.^11^ Sepsis was defined by two or more of the following conditions as a result of infection: (a) temperature >38^°^ or <36^°^, (b) heart rate >90 beats per minute, (c) respiratory rate >20 breaths per minute or PaCO_2_ <32 mm Hg, and (d) WBC count >12 000 cells/mm^3^ or <4000 cells/mm^3^.^12^

### 2.3 Statistical analyses

Baseline characteristics, laboratory findings, and clinical outcomes were compared according to DNI values and clinical parameters. Continuous variables were compared using Student's *t* test, and categorical variables were compared with chi‐square test or Fisher's exact test. All *P‐*values <0.05 were considered statistically significant. Cutoff values of prothrombin time, aPTT, CRP, creatinine, albumin, and DNI were obtained from receiver operating characteristic (ROC) curves drawn for each group in relation to 30‐day mortality. SPSS software version 22.0 (SPSS, Chicago, IL, USA) was used for all statistical analyses.

## 3 RESULTS

### 3.1 Comparison of clinical characteristics and laboratory findings in patients with acute peritonitis (Group I) between 30‐day mortality and non‐mortality groups

Among 11 patients with 30‐day mortality after emergent surgery, 10 patients were included in the group of acute peritonitis. Clinical and laboratory findings were compared by dividing patients into either a 30‐day mortality group or a non‐mortality group (Table 1). Age and gender were not significantly different in the two groups. However, patients with underlying diabetes and cardiac disease were more frequent in the mortality group than those in the non‐mortality group. Patients who exhibited bacteremia (40.0% vs 1.1%, *P* < 0.001) and sepsis (50.0% vs 17.8%, *P* = 0.026) were significantly higher in the mortality group. Postoperative hospital stay was not significantly different between the two groups (12.6 days vs 10.2 days, *P* = 0.436). However, postoperative ICU stay of the mortality group was significantly longer than that of the non‐mortality group (8.2 days vs 1.0 days, *P* = 0.018).

**Table 1:** Comparison of clinical and laboratory findings in patients with acute peritonitis between 30‐d mortality and non‐mortality groups

Variables	Mortality (n = 10)	Non‐mortality (n = 174)	*P*‐Value
Age, years, mean ± SD	59.1 ± 20.7	53.5 ± 18.1	0.308
Male sex, n (%)	5 (41.7)	113 (65.7)	0.121
Comorbidity			
HTN, n (%)	5 (50.0)	40 (23.0)	0.066
DM, n (%)	4 (40.0)	14 (8.0)	0.009
Cardiac disease, n (%)	4 (40.0)	3 (1.7)	<0.001
Renal disease, n (%)	0 (0.0)	1 (0.6)	1.000
Malignancy, n (%)	0 (0.0)	19 (10.9)	0.602
Laboratory findings			
WBC, ×10^3^/µL, mean ± SD	12.64 ± 7.64	11.47 ± 4.60	0.456
Neutrophils, %, mean ± SD	75.92 ± 14.33	80.31 ± 10.99	0.229
Absolute neutrophil count, ×10^3^/µL, mean ± SD	9.96 ± 6.64	9.43 ± 4.35	0.717
Platelet, ×10^3^/µL, mean ± SD	202.40 ± 54.75	246.81 ± 82.36	0.094
Prothrombin time, INR, mean ± SD	1.26 ± 0.18	1.08 ± 0.11	0.015
aPTT, sec, mean ± SD	43.11 ± 7.67	36.98 ± 5.17	0.033
CRP, mg/L, mean ± SD	94.43 ± 63.01	44.17 ± 48.35	0.002
Creatinine, mg/dL, mean ± SD	1.99 ± 1.06	0.90 ± 0.31	0.010
Albumin, g/dL, mean ± SD	3.00 ± 0.91	4.00 ± 0.50	0.007
DNI, %, mean ± SD	4.20 ± 3.50	1.46 ± 2.41	0.036
Bacteremia, n (%)	4 (40.0)	2 (1.1)	<0.001
Sepsis, n (%)	5 (50.0)	31 (17.8)	0.026
Postoperative hospital stay, days, mean ± SD	12.6 ± 12.4	10.2 ± 9.3	0.436
Postoperative ICU stay, days, mean ± SD	8.2 ± 7.9	1.0 ± 2.3	0.018

aPTT, activated partial thromboplastin time; CRP, C‐reactive protein; DM, diabetes mellitus; DNI, delta neutrophil index; HTN, hypertension; ICU, intensive care unit.; SD, standard deviation; WBC, white blood cell. Bold defined that *P*‐value of variables was < 0.05.

In laboratory findings, WBC and platelet counts were not significantly different between the two groups. Prothrombin time, aPTT, CRP, and creatinine of the mortality group were significantly higher than those of the non‐mortality group while albumin was significantly lower in the mortality group. The DNI value was significantly higher in the mortality group than that in the non‐mortality group (4.20% vs 1.46%, *P* = 0.036). Bold defined that *P*‐value of variables was < 0.05.

### 3.2 Receiver operating characteristic analysis for predicting 30‐day mortality in Group I

**Figure 2:**
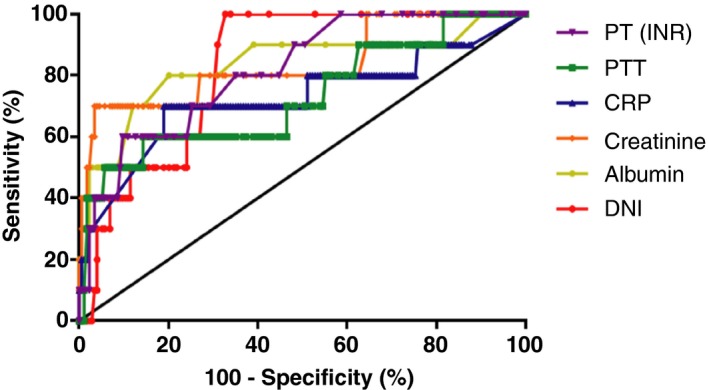
Receiver operating characteristic curves comparing the ability of prothrombin time, partial thromboplastin time, C‐reactive protein, creatinine, albumin, and delta neutrophil index according to 30‐d mortality

We evaluated the relationship between 30‐day mortality and laboratory findings including prothrombin time, aPTT, CRP, creatinine, albumin, and DNI. These values were statistically significant in patients with acute peritonitis. In ROC curve analysis, predictive values of aPTT and CRP for 30‐day mortality were only fair (area under the curve [AUC]: 0.729 and 0.727, respectively) (Table 2). On the other hand, predictive values of prothrombin time, creatinine, albumin, and DNI were good (AUC: 0.816, 0.837, 0.834, and 0.826, respectively). The best cutoff level of DNI for the prediction of 30‐day mortality in acute peritonitis was 0.9 or greater with a sensitivity of 100.0% and a specificity of 67.2%. ROC curves using variables are plotted in Figure 2.

**Table 2:** Values of ROC curves according to 30‐d mortality for the prothrombin time, partial thromboplastin time, CRP, creatinine, albumin, and DNI in acute peritonitis

Variables	AUC (95% CI)	Sensitivity, % (95% CI)	Specificity, % (95% CI)	Cutoff level
Prothrombin time	0.816 (0.693‐0.938)	60.0 (26.2‐87.8)	90.2 (84.8‐94.2)	1.22
aPTT	0.729 (0.545‐0.914)	60.0 (26.2‐87.8)	85.6 (79.5‐90.5)	42.90
CRP	0.727 (0.525‐0.930)	70.0 (34.8‐93.3)	81.0 (74.4‐86.6)	111.80
Creatinine	0.837 (0.680‐0.993)	70.0 (34.8‐93.3)	96.6 (92.7‐98.7)	1.50
Albumin	0.834 (0.673‐0.994)	80.0 (44.4‐97.5)	79.9 (73.2‐85.6)	3.65
DNI	0.826 (0.741‐0.911)	100.0 (69.2‐100.0)	67.2 (59.7‐74.2)	0.90

aPTT, activated partial thromboplastin time; CI, confidence interval; CRP, C‐reactive protein; DNI, delta neutrophil index.

### 3.3 Comparison of clinical characteristics and laboratory findings between patients with high and low DNI

We divided subjects into two groups according to whether their DNI level was 0.9% or greater or <0.9%. Table 3 shows comparison data between DNI‐high and DNI‐low groups. Patients with high DNI values were older with more male patients. They had higher comorbidity of diabetes, cardiac disease, and malignancy than patients with DNI‐low values. According to laboratory findings, WBC, neutrophils, absolute neutrophil count, prothrombin time, CRP, and creatinine were higher in the DNI‐high group than those in the DNI‐low group while albumin was lower in the DNI‐high group. Among 305 patients diagnosed with acute non‐perforated appendicitis, 70 (23.0%) had DNI value of 0.9% or greater. In the patients with acute cholecystitis, 61 (29.8%) were included in the DNI‐high group. More patients had bacteremia and sepsis in the DNI‐high group than those in the DNI‐low group. Also, patients with high levels of DNI exhibited longer postoperative hospital (6.6 days vs 5.0 days, *P* = 0.010) and ICU stays (1.1 days vs 0.2 days, *P* < 0.001) than those with low levels of DNI. Postoperative complications occurred in 12 patients (five patients with abscess, three patients with wound infection, three patients with ileus, and one patient with pneumonia) of the DNI‐high group and two patients (one patient with abscess and one patient with wound infection) of the DNI‐low group (*P* < 0.001). The 30‐day mortality was higher in the DNI‐high group than that in the DNI‐low group (5.1% vs 0.2%, *P* < 0.001).

**Table 3:** Comparison of clinical characteristics and laboratory findings between patients in DNI‐High and DNI‐Low groups

Variables	DNI‐High (n = 198)	DNI‐Low (n = 496)	*P*‐Value
Age, years, mean ± SD	53.0 ± 17.6	46.9 ± 17.6	<0.001
Male sex, n (%)	122 (61.6)	252 (50.8)	0.010
Comorbidity			
HTN, n (%)	49 (24.7)	95 (19.2)	0.101
DM, n (%)	25 (12.6)	38 (7.7)	0.040
Cardiac disease, n (%)	14 (7.1)	13 (2.6)	0.006
Renal disease, n (%)	2 (1.0)	4 (0.8)	1.000
Malignancy, n (%)	16 (8.1)	21 (4.2)	0.042
Laboratory findings			
WBC, ×10^3^/µL, mean ± SD	13.11 ± 5.10	11.52 ± 4.06	<0.001
Neutrophils, %, mean ± SD	84.07 ± 8.51	77.54 ± 11.05	<0.001
Absolute neutrophil count, ×10^3^/µL, mean ± SD	11.19 ± 4.77	9.19 ± 3.98	<0.001
Platelet, ×10^3^/µL, mean ± SD	228.58 ± 70.83	235.07 ± 67.12	0.258
Prothrombin time, INR, mean ± SD	1.10 ± 0.13	1.06 ± 0.14	0.002
aPTT, sec, mean ± SD	38.00 ± 7.05	37.20 ± 4.85	0.142
CRP, mg/L, mean ± SD	46.69 ± 53.51	26.26 ± 38.43	<0.001
Creatinine, mg/dL, mean ± SD	1.00 ± 0.73	0.86 ± 0.51	0.018
Albumin, g/dL, mean ± SD	3.98 ± 0.53	4.13 ± 0.42	0.001
Diagnosis			
Acute peritonitis, n (%)	67 (33.8)	117 (23.6)	0.178
Acute non‐perforated appendicitis, n (%)	70 (35.4)	235 (47.4)	0.178
Acute cholecystitis, n (%)	61 (30.8)	144 (29.0)	0.178
Bacteremia, n (%)	18 (9.1)	16 (3.2)	0.001
Sepsis, n (%)	52 (26.3)	51 (10.3)	<0.001
Postoperative hospital stay, days, mean ± SD	6.6 ± 8.2	5.0 ± 4.9	0.010
Postoperative ICU stay, days, mean ± SD	1.1 ± 3.2	0.2 ± 1.0	<0.001
Complication, n (%)	12 (6.1)	2 (0.4)	<0.001
The 30‐d mortality, n (%)	10 (5.1)	1 (0.2)	<0.001

aPTT, activated partial thromboplastin time; CRP, C‐reactive protein; DM, diabetes mellitus; DNI, delta neutrophil index; HTN, hypertension; ICU, intensive care unit.; SD, standard deviation; WBC, white blood cell. Bold defined that *P*‐value of variables was < 0.05.

### 3.4 Comparison of clinical characteristics and laboratory findings between patients with acute perforated appendicitis and those with non‐perforated appendicitis

Clinical characteristics and laboratory findings were compared between 78 patients diagnosed with acute perforated appendicitis and 305 patients with acute non‐perforated appendicitis (Table 4). Patients with acute perforated appendicitis were older and male‐dominant. They had more frequent diabetes than patients with acute non‐perforated appendicitis. The postoperative hospital stay of the perforated group was significantly longer than that of the non‐perforated group (5.1 days vs 2.9 days, *P* < 0.001). Only two patients of the perforated group required a postoperative ICU stay. The incidence rate of postoperative complications of the perforated group (three patients: one patient with abscess, one patient with wound infection, and one patient with ileus) was not significantly different from that of the non‐perforated group (six patients: three patients with abscess, two patients with wound infection, and one patient with ileus) (3.8% vs 2.0%, *P* = 0.396). The 30‐day mortality was not significant in either group.

**Table 4:** Comparison of clinical and laboratory findings between patients with acute perforated appendicitis and those with non‐perforated appendicitis

Variables	Perforated (n = 78)	Non‐perforated (n = 305)	*P*‐Value
Age, years, mean ± SD	45.3 ± 15.3	38.3 ± 12.8	<0.001
Male sex, n (%)	50 (64.1)	152 (49.8)	0.030
Comorbidity			
HTN, n (%)	10 (12.8)	25 (8.2)	0.269
DM, n (%)	6 (7.7)	3 (1.0)	0.003
Cardiac disease, n (%)	0 (0.0)	3 (1.0)	1.000
Renal disease, n (%)	1 (1.3)	1 (0.3)	0.366
Malignancy, n (%)	2 (2.6)	4 (1.3)	0.354
Laboratory findings			
WBC, ×10^3^/µL, mean ± SD	12.93 ± 4.46	13.10 ± 3.90	0.736
Neutrophils, %, mean ± SD	82.68 ± 9.10	80.74 ± 8.85	0.087
Absolute neutrophil count, ×10^3^/µL, mean ± SD	10.87 ± 4.24	10.78 ± 3.82	0.885
Platelet, ×10^3^/µL, mean ± SD	232.05 ± 57.16	234.59 ± 53.56	0.712
Prothrombin time, INR, mean ± SD	1.08 ± 0.09	1.04 ± 0.06	<0.001
aPTT, sec, mean ± SD	38.75 ± 5.19	36.91 ± 3.91	0.004
CRP, mg/L, mean ± SD	60.46 ± 61.97	30.67 ± 42.70	0.010
Creatinine, mg/dL, mean ± SD	0.89 ± 0.29	0.88 ± 1.14	0.956
Albumin, g/dL, mean ± SD	4.14 ± 0.34	4.24 ± 0.34	0.018
DNI, %, mean ± SD	1.36 ± 1.99	0.72 ± 1.10	0.008
Bacteremia, n (%)	1 (1.3)	0 (0.0)	0.204
Sepsis, n (%)	16 (20.5)	37 (12.1)	0.066
Postoperative hospital stay, days, mean ± SD	5.1 ± 2.2	2.9 ± 2.0	<0.001
Postoperative ICU stay, n (%)	2 (2.6)	0 (0.0)	0.041
Complication, n (%)	3 (3.8)	6 (2.0)	0.396
The 30‐d mortality, n (%)	0 (0.0)	0 (0.0)	1.000

aPTT, activated partial thromboplastin time; CRP, C‐reactive protein; DM, diabetes mellitus; DNI, delta neutrophil index; HTN, hypertension; ICU, intensive care unit.; SD, standard deviation; WBC, white blood cell. Bold defined that *P*‐value of variables was < 0.05.

In laboratory findings, WBC and platelet counts were not significantly different between the perforated and non‐perforated groups. Prothrombin time, aPTT, CRP, and albumin of the perforated group were significantly different from those of the non‐perforated group. DNI value of the perforated group was higher than that of the non‐perforated group (1.36% vs 0.72%, *P* = 0.008).

## 4 DISCUSSION

The present study demonstrated that DNI value was correlated with severe infection and poor prognosis in patients with acute abdomen. Patients with high levels of DNI (≥0.9%) displayed higher incidence of bacteremia and sepsis, longer hospital and ICU stay, and higher rate of postoperative complications than patients with low DNI levels (<0.9%). In addition, 30‐day mortality was higher in patients with high DNI values. In acute peritonitis, DNI could predict 30‐day mortality. It was not inferior to other laboratory markers associated with infection. In acute appendicitis, DNI value was a useful marker for appendiceal perforation. Our results indicated that DNI could be a useful tool for predicting severity and prognosis in acute abdomen.

According to recent studies, DNI is a predictive marker of histological chorioamnionitis in patients with preterm premature rupture of membranes.^13^ A higher DNI is a prognostic marker of out‐of‐hospital cardiac arrest^14^ and an independent factor of mortality in septic acute kidney injury patients with continuous renal replacement therapy.^15^ Septic condition of patients who visited the emergency room is an important factor for predicting their prognosis and mortality. Therefore, there have been efforts to find proper biomarkers associated with sepsis. The utility of DNI value in patients with sepsis and bacteremia has been reported in several studies.^6^, ^7^, ^8^, ^16^, ^17^ In a previous study, the DNI value was used as an early marker of disease severity in critically ill patients with sepsis.^18^ However, in another study, the use of DNI for predicting bacteremia or sepsis was limited to immunocompromised cases.^19^ Recently, a meta‐analysis was performed for infected patients to confirm whether DNI could function as a reliable parameter.^20^ It demonstrated that DNI was a potentially useful diagnostic tool in diagnosing infection and predicting mortality. In the present study, patients with acute abdomen were targeted. Patients with DNI value of more than 0.9% when arriving at the emergency room had longer postoperative hospital stay, higher mortality, and higher rates of sepsis, bacteremia, and postoperative complications than those with DNI value of below 0.9%, although these patients were older with more comorbidities. These results supported a practical value of DNI as prognostic marker for infectious diseases of the abdomen.

Initial DNI level and myeloperoxidase index as diagnostic predictors of strangulated mechanical bowel obstruction in emergency setting have been reported,^21^ consistent with finding of the present study. Acute peritonitis was the primary disease requiring abdominal emergent surgery. To the best of our knowledge, this study assessed the utility of DNI in patients with acute peritonitis for the first time. DNI can be provided with routine CBC that is performed necessarily upon arrival at the emergency department. With other serologic markers including CRP, prothrombin time, aPTT, creatinine, and albumin associated with infection, DNI significantly predicted death within 30 days after abdominal surgery. In addition, an AUC value of DNI at a cutoff level of 0.9% displayed better accuracy than that of aPTT or CRP in Group I. Patients with acute peritonitis require an examination of the DNI value as a prognostic factor.

Studies for the role of DNI in differentiating perforated appendicitis from non‐perforated appendicitis have been conducted in the elderly^10^ and children.^22^ In previous studies, the predictive value of the DNI for complicated appendicitis was good with an AUC of 0.807 in the elderly and fair with an AUC of 0.738 in children. The present study demonstrated a significant difference in DNI values between patients with perforated and those with non‐perforated appendicitis. However, DNI had a poor predictive value with an AUC of 0.623 for differentiating appendiceal perforation in ROC analysis. At a cutoff level of 1.45%, its sensitivity and specificity were 32.1% and 85.3%, respectively. In the general population of those aged 18 years and older, the result of this study was different from those of previous studies. Further study with a large number of subjects is needed to confirm the role of DNI in predicting appendiceal perforation.

The first limitation of the present study was that it was retrospective in nature with subjects from in a single center. Such limitation might have resulted in selection bias. Second, many infectious diseases of the abdomen were included in the group of acute peritonitis. Because the severity and activity were diverse for these diseases, assessments of acute peritonitis might not have been consistent. Third, DNI was only measured upon arrival at the emergency room. Serial changes in DNI values according to aggravation or improvement of the infection were not examined in this study. Fourth, other inflammatory serology markers such as ESR or procalcitonin were not evaluated in the present study. These markers were not routinely checked in our hospital.

In conclusion, DNI is a valuable prognostic marker in patients who visited the emergency room complaining of acute abdominal pain. Patients with DNI level of 0.9% or greater who needed emergent abdominal surgery or required surgical intervention for acute peritonitis should be monitored closely with appropriate treatment strategies. DNI could be helpful for selecting high‐risk patients and deciding therapeutic modalities such as emergent operation or intensive care unit treatment.
